# Ambiguity and variability of database and software names in bioinformatics

**DOI:** 10.1186/s13326-015-0026-0

**Published:** 2015-06-29

**Authors:** Geraint Duck, Aleksandar Kovacevic, David L. Robertson, Robert Stevens, Goran Nenadic

**Affiliations:** School of Computer Science, The University of Manchester, Oxford Road, Manchester, M13 9PL UK; Faculty of Technical Sciences, University of Novi Sad, Novi Sad, Serbia; Computational and Evolutionary Biology, Faculty of Life Sciences, The University of Manchester, Oxford Road, Manchester, M13 9PT UK; Manchester Institute of Biotechnology, The University of Manchester, 131 Princess Street, Manchester, M1 7DN UK

**Keywords:** Bioinformatics, Computational biology, CRF, Dictionary, Resource extraction, Text-mining

## Abstract

**Background:**

There are numerous options available to achieve various tasks in bioinformatics, but until recently, there were no tools that could systematically identify mentions of databases and tools within the literature. In this paper we explore the variability and ambiguity of database and software name mentions and compare dictionary and machine learning approaches to their identification.

**Results:**

Through the development and analysis of a corpus of 60 full-text documents manually annotated at the mention level, we report high variability and ambiguity in database and software mentions. On a test set of 25 full-text documents, a baseline dictionary look-up achieved an F-score of 46 %, highlighting not only variability and ambiguity but also the extensive number of new resources introduced. A machine learning approach achieved an F-score of 63 % (with precision of 74 %) and 70 % (with precision of 83 %) for strict and lenient matching respectively. We characterise the issues with various mention types and propose potential ways of capturing additional database and software mentions in the literature.

**Conclusions:**

Our analyses show that identification of mentions of databases and tools is a challenging task that cannot be achieved by relying on current manually-curated resource repositories. Although machine learning shows improvement and promise (primarily in precision), more contextual information needs to be taken into account to achieve a good degree of accuracy.

## Background

Bioinformatics and computational biology rely on domain databases and software to support data collection, aggregation and analysis and, as such, have been reported in research papers, typically as part of the methods section. However, limited progress has been made to systematically capture mentions of databases and tools in order to explore the bioinformatics practice of computational method on a large-scale. An evaluation of the resources available could help bioinformaticians to identify common usage patterns [1] and potentially infer scientific “best practice” [2] based on a measure of how often or where a particular resource is currently being used within an *in silico* workflow [3]. Although there are several inventories that list available database and software resources (e.g., the NAR databases and web-services special issues [4, 5], ExPASy [6], the Online Bioinformatics Resources Collection [7], etc.), until recently, to the best of our knowledge, there were no attempts to systematically identify resource mentions in the literature [8].

Biomedical text mining has seen wide usage in identifying mentions of entities of different types in the literature in recent years. Named entity recognition (NER) enables automated literature insights [9] and provides input to other text-mining applications. For example, within the fields of biology and bioinformatics, NER systems have been developed to capture species [10], proteins/genes [11–13], chemicals [14], etc. Issues of naming inconsistencies, numerous synonyms and acronyms, and an inability to distinguish entity names from common words in a natural language combined with ambiguous definitions of concepts, make NER a difficult task [15, 16]. Still, for some applications, NER tools achieve relatively high precision and recall scores. For example, LINNAEUS achieved F-scores around the 95 % mark for species name recognition and disambiguation on the mention and document levels [10]. On the other hand, gene names are known for their ambiguity and variability, resulting in lower reported F-scores. For example, ABNER [12] recorded an F-score of just under 73 % for strict-match gene name recognition (85 % with some boundary error toleration), and GNAT [13] reported an F-score of 81 % for the same task (up to a maximum of 90 % for single species gene name recognition, e.g., for yeast).

Some previous work exists on automated identification and harvesting of bioinformatics database and software names from the literature. For example, OReFiL [17] utilises the mentions of Unified Resource Locators (URLs) in text to recognise new resources to update its own internal index. Similarly, BIRI (BioInformatics Resource Inventory) uses a series of hand crafted regular expressions to automatically capture resource names, their functionality and classification from paper titles and abstracts [18]. The reported quality of the identification process was in line with other NER tasks. For example, BIRI successfully extracted resource names in 94 % of cases in a test corpus, which consisted of 392 abstracts that matched a search for “bioinformatics resource” and eight documents that were manually included to test domain robustness. However, both of these tools focused on updates and have biased their evaluation to resource rich text, which prevents full understanding of false negative errors in the general bioinformatics literature.

This paper aims to analyse database and software name mentions in the bioinformatics/computational biology literature to assess challenges for automated extraction. We analyse database and software names in the computational biology literature using a set of 60 full-text documents manually annotated at the mention level, building on our previous work [19]. We analyse the variability and ambiguity of bioinformatics resource names and compare dictionary and machine learning approaches for their identification based on the results on an additional dataset of 25 full-text documents. Although we focus here on bioinformatics resources, the challenges and solutions encountered in database and software recognition are generic, and thus not unique to this domain [20].

## Methods

### Corpus annotation and analysis

For the purpose of this study, we define *databases* as any electronic resource that stores records in a structured form, and provides unique identifiers to each record. These include any database, ontology, repository or classification resource, etc. Examples include *SCOP* (a database of protein structural classification) [21], *UniProt* (a database of protein sequences and functional information) [22], *Gene Ontology* (ontology that describes gene product attributes) [23], *PubMed* (a repository of abstracts) [24], etc. We adopt Wikipedia’s definition of *software* [25]: “a collection of computer programs … that provides the instructions for telling a computer what to do and how to do it”. We use *program* and *tool* as synonyms for software. Examples include *BLAST* (automated sequence comparison) [26], *eUtils* (access to literature data) [27], etc. We also include mentions of web-services as well as package names (e.g., *R* packages from *Bioconductor* [28, 29]). We explicitly exclude database record numbers/identifiers (e.g., *GO:0002474*, *Q8HWB0*), file formats (e.g., *PDF*), programming languages and their libraries (e.g., *Python*, *BioPython*), operating systems (e.g., *Linux*), algorithms (e.g., *Merge-Sort*), methods (e.g., *ANOVA*, *Random Forests*) and approaches (e.g., *Machine Learning*, *Dynamic Programming*).

To explore the use of database and tool names, we have developed an annotated set of 60 full-text articles from the PubMed Central [30] open-access subset. The articles were randomly selected from Genome Biology (5 articles), BMC Bioinformatics (36) and PLoS Computational Biology (19). These journals were selected as they could provide a broad overview of the bioinformatics and computational biology domain(s).

The articles were primarily annotated by a bioinformatician (GD) with experience in text mining. The annotation process included marking each database/software name mention. We note that associated designators of resources (e.g., words such as *database*, *software*) were included only if part of the official name (e.g., *Gene Ontology*). The inter-annotator agreement (IAA) [31] for the annotation of database and software names was calculated from five full-text articles randomly selected from the annotated corpus, which were annotated by a PhD student with bioinformatics and a text-mining background.

To assess the complexity, composition, variability and ambiguity of resource names, we performed an analysis of the annotated mentions. The corpus was pre-processed using a typical text-mining pipeline consisting of a tokeniser, sentence splitter and part-of-speech (POS) tagger from GATE’s ANNIE [32]. We analysed the length of names, their lexical (stemmed token-level) and structural composition (using POS tag patterns) and the level of variability and ambiguity as compared to common English words, acronyms and abbreviations.

In addition to the dataset of 60 articles that was used for analysis and development of NER tools, an additional dataset of 25 full-text annotated papers was created to assess the quality of the proposed NER approaches (see below).

### Dictionary-based approach (baseline)

We compiled an extensive dictionary of database and software names from several existing sources (see Table 1). Some well-known acronyms and spelling/orthographic variants have also been added, resulting in 7322 entries with 8169 variants (6929 after removing repeats) for 6126 resources. The names collected in the dictionary were also analysed using a similar approach as used for the names appearing in the corpus (see above). We then used LINNAEUS [10] to match these names in text.

**Table 1:** Sources from which the database and software name dictionary is comprised

Type	Entries	Variants	Source
DB	195	298	databases.biomedcentral.com
SW	263	278	www.bioinformatik.de
PK	799	799	www.bioconductor.org
SW	2033	2087	bioinformatics.ca/links_directory/
SW	389	391	evolution.genetics.washington.edu/phylip/software.html
DB	379	379	www.ebi.ac.uk/miriam/main/
DB	1452	1670	www.oxfordjournals.org/nar/database/a/
SW	135	135	www.netsci.org/Resources/Software/Bioinform/index.html
SW	36	41	www.bioinf.manchester.ac.uk/recombination/programs.shtml
SW	1149	1183	en.wikipedia.org/wiki/Wiki/<*various*>
SW, DB	171	231	Manually added entries
Our dictionary (DB, SW, PK)	7322	6929	http://sourceforge.net/projects/bionerds/

Note that entries and variants are not necessarily unique to a single resource list

*DB* databases, *SW* software, *PK* packages; data correct and accessible as of February 28th, 2012

### Machine learning approach

Given the availability of the manually annotated corpus, a machine learning (ML) approach was explored for identification of resource names. We approached the task as a sequence-tagging problem as often adopted in NER systems. We opted for Conditional Random Fields (CRF) [33] and used features at the token-level that comprised the token’s own characteristics and the features of the neighbouring tokens. We used the Beginning-Inside-Outside (B-I-O) annotation.

The following features were engineered for each token:

1. **Orthographic features** captured the orthographic patterns associated with biomedical resources mentions. For example, a large percentage of mentions are acronyms (e.g., *GO*, *SCOP*), capitalised terms (e.g., *Gene Ontology*, *Bioconductor*) or words that contain a combination of capital and lower cap letters (e.g., *MySQL*, *UniProt*) etc. We engineered two groups of orthographic features [34]. The first group comprised shape (pattern) features that mapped a given token to an abstract representation. Each capital letter is replaced with “X”, lower case letter with “x”, a digit with “d” and any other character with “S”. Two features were created in this group: the first feature contained a mapping for each character in a token (e.g., *MySQL* was mapped to “XxXXX”); the second feature mapped a token to a four character string that contained indicators of a presence of a capital letter, a lower letter, a digit or any other character (absence was mapped to a “_”), e.g., *MySQL* was mapped to “Xx_ _”. The features in the second group captured specific orthographic characteristics (e.g., is the token capitalised, does it consist of only capital letters, does it contain digits, etc. – see Table 2 for the full list), which were extracted by a set of regular expressions.

**Table 2:** Token-specific orthographic features extracted by regular expressions

Name	Description
isAcronym	token is an acronym
containsAllCaps	all the letters in the token are capitalised
isCapitalised	token is capitalised
containsCapLetter	token contains at least one capital letter
containsDigits	token contains at least one digit
isAllDigits	token is made up of digits only
2. **Dictionary features** were represented by a single binary feature that indicated if the given token was contained within our biomedical resources dictionary.
3. **Lexical features** included the token itself, its lemma and part-of-speech (POS) tag.
4. **Syntactic features** were extracted from syntactic relations in which the phrase was a *governor* or a *dependant*, as returned by the Stanford parser [35, 36]; in cases where there were several relations, the relation types were alphabetically sorted and concatenated (e.g., “pobj” and “advmod” were combined as “advmod_pobj”).

The experiments on the training data revealed that two tokens before and one token after the current token provided the best performance. The CRF model was trained using CRF++ [37]. All pre-processing needed for feature extraction was provided by the same text-mining pipeline as used for the corpus analysis and dictionary-based approach.

### Machine learning – post-processing

An analysis of the initial CRF results on the development dataset revealed that a large portion of false negatives were from resource mentions that were recognised by the model at least once in a document, but missed elsewhere within the same document. We have therefore designed a two-pass post-processing approach. The first pass collected and stored all the CRF tagging results. These were then used to re-label the tokens in the second pass. In order to avoid over-generation of labels (i.e., possible false positives), we created a set of conditions that each token had to meet if it was to be re-labelled as a resource mention. First, it had to be labelled as a (part of a) resource name in the first pass more often than it was not, looking at the entire corpus that was being tagged. If that was the case, the candidate token also had to fulfil one of the following two conditions: either it was contained within the biomedical resources dictionary; or it was an acronym that had no digits and was at least two characters long. Finally, the following four tokens: “analysis”, “genomes”, “cycle” and “cell” were never labelled as part of resource name in the second round, as they were found to be the source of a large percentage of false positives.

### Evaluation

Standard text-mining performance statistics (precision, recall, F-score) were used for evaluation. In particular, we make use of 5-fold cross-validation across all 60 full-text articles for both the dictionary and machine learning approaches. For a fair comparison, the dictionary-based approach is only evaluated on the test set in each fold, as it requires no prior “training”. We also test both approaches directly on the test set of 25 articles without additional training/adjustments.

## Results and discussion

### Corpus annotations

Table 3 gives an overview of the two corpora annotated with resource mentions. We note that the IAA was reasonably high: with lenient agreement (annotation offsets overlap), an F-score of 86 % was calculated (93 % precision, 80 % recall). As expected, a decrease in IAA is observed if strict agreement (offsets must exactly match) is used instead (every score drops by 6 %).

**Table 3:** Statistics describing the manually annotated corpora

	Development	Test
Total number of documents	60	25
Total database and software mentions	2416	1479
Total unique resource mentions	401	301
Percentage of database mentions	36 %	28 %
Percentage of unique database mentions	27 %	30 %
Average mentions per document	40.3	70.0
Average unique mentions per document	8.1	13.4
Maximum mentions in a single document	227	217
Maximum unique mentions in a single document	57	55
Resources with only a single lexicographic mention	201	147

In the development corpus, there were 401 lexically unique resources mentioned 2416 times (6 mentions on average per unique resource name), with an average of 40 resource mentions per document. The document with the most mentions had 227 resource mentions within it. Finally, 50 % of resource names were only mentioned once in the corpus. A similar profile was noted for the test corpus, although it contained notably more resource mentions per document.

### Database and software name composition

We first analysed the composition of resource names both in the development corpus and dictionary. The longest database/software name in the annotated corpus contained ten tokens (i.e., *Search Tool for the Retrieval of Interacting Genes/Proteins*). However, there are longer examples in the dictionary (e.g., *Prediction of Protein Sorting Signals and Localisation Sites in Amino Acid Sequences*).

**Fig. 1:**
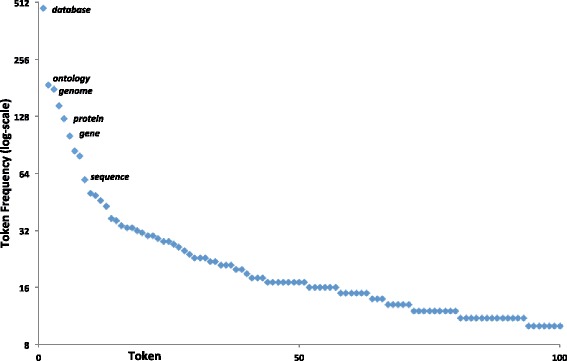
Top token frequencies within the manually compiled dictionary. The figure shows the most common stemmed tokens contained within all the resource names found within our manually compiled dictionary. The top token is *database* with a count of 474, followed by *ontology* with 187 instances. Note that the scale is logarithmic (log base 2), and the y-axis crosses at eight rather than zero (for aesthetic reasons). The top terms are labelled

To assess the composition of resource names within our dictionary, we stemmed each token within each name (using the Porter Stemming Algorithm [38]) and counted the occurrences of each stemmed token. Figure 1 displays the most frequent words: the two most ones are *database* and *ontology*. A comparable lexical distribution can be noted in the development set, with *database, gene, analysis, tool, genome, ontology* featuring as the most frequent ones (data not shown). This suggests that some common *head* terms and some other common bioinformatics relevant terms could aid recognition. We also note that there is a long tailed curve involved in the lexical decomposition of resource words.

As an initial structural analysis, we automatically collected all the POS tags assigned to each unique database and software name in the development corpus. These were then grouped to profile the structure of resource names (see Table 4). We have identified a total of 405 patterns. The majority (79 %) of database and software names comprise one, two or three proper nouns. An additional 5 % were tagged a single common noun (e.g., *affy*). A roughly equivalent number of names contain digits (e.g., *S4*, *t2prhd*). Nine patterns contain adjectives (e.g., *internal transcribed spacer 2*) or prepositions/subordinating conjunctions (e.g., *Structural Classification Of Proteins*). Finally, in two cases (*SHAKE* and *dot*), a mention of software was tagged as a verb form. We note that there are more patterns (405) than unique mentions (401) because sometimes an equal resource name gets tagged with differing patterns (e.g., *R* received both NNP and NN POS tags). The analysis shows that there is some variety in resource naming, and – as expected – that recognition of simple noun phrases alone is not sufficient for identification of potential resource mentions. In particular, around 5 % of noun-phrases (as extracted with the Stanford Parser) within the corpus contain at least one resource mention.

**Table 4:** Internal POS structure of database and software names (the development corpus)

Pattern	Count	Frequency
NNP	258	63.7 %
NNP NNP	34	8.4 %
NNP NNP NNP	26	6.4 %
NN	20	4.9 %
NNP CD	16	4.0 %
NNP NNP NNP NNP	8	2.0 %
Other Patterns	43	10.6 %

*NNP* proper noun, *NN* singular noun, *CD* cardinal number

### Variability of resource names

To evaluate the variability of resource names within our dictionary, we calculated the average number of name variants for a given resource. As such, the variability of resource names at the dictionary level is 1.13 (6929 unique variants over 6126 resources, after adjustment for repeats). For the corpus analysis, we manually grouped the names from the set of manually annotated mentions that were referring to the same resource in order to analyse name variability. Specifically, we grouped variants based on spelling errors and orthographic differences, and then grouped long and short form acronym pairs based on our own background knowledge, and the text from which they were initially extracted. Of the 401 lexically unique names, 97 were variants of other names, leaving 304 unique resources. In total, 231 resources had only a single name variant within the corpus (76 %); 18 % of resources had two variants, and the final 6 % had between three and five variants. Of the 97 name variants, 36 were acronyms and most of those were defined in text (and so could perhaps be automatically expanded with available tools, e.g., [39]). However, there were other cases where a resource’s acronym was used without the expanded form for definition (e.g., *BLAST*).

### Ambiguity of resource names

As expected, a number of ambiguous resource names exist within the bioinformatics domain. Interesting examples include *Network* [40] (a tool enabling network inference from various biological datasets) and *analysis* [41] (a package for DNA sequence analysis). We therefore analysed our dictionary of database and software names to evaluate dictionary-level ambiguity when compared to the entries in a full English words dictionary derived from a publicly available list [42] (hereafter referred to as the “English dictionary”) and to a known biomedical acronyms dictionary compiled from ADAM [43] (hereafter referred to as the “acronym dictionary”), consisting of 86,308 and 1933 terms, respectively. A total of 52 names matched English words (e.g., *analysis*, *cycle*, *graph*) and 77 names fully matched known acronyms (e.g., *DIP*, *distal interphalangeal* and *Database of Interacting Proteins*) when using case-sensitive matching. The number of matches increases to 534 to the English dictionary and to 96 for the acronym dictionary when case-insensitive matching is used instead.

To evaluate the recognition-level ambiguity within the annotated corpus, we also compared the annotated database and software names to the English dictionary and acronym dictionary. This resulted in four matches to the English dictionary (*ACT*, *blast*, *dot*, *R*), and six to the acronym dictionary (*BBB*, *CMAP*, *DIP, IPA, MAS, VOCs*) using case-sensitive matching. This equates to roughly 3 % of the unique annotated names. The total increases to 53 matches (17 %) if case-insensitive matching is used instead.

### Dictionary-based matching

Table 5 provides the standard text-mining performance statistics for the dictionary matching approach. The average lenient F-scores between 43 and 46 % highlight the challenges for this approach, both in terms of matching known ambiguous names (low precision), and from the dictionary not being sufficiently comprehensive (low recall). Some common false positives were *cycle*, *genomes* (potential mentions of *Bioconductor* packages) and *GO* (which was frequently matched within *GO* database identifiers (e.g., *GO:0007089*) because of inappropriate tokenisation). Some common false negatives (i.e., missed resource mentions) included *Tabasco* (PMC2242808), *MethMarker* (PMC2784320), *xPedPhase* and *i Linker* (both from PMC2691739). In each of these examples, the name missed (numerous times) was the resource being introduced in that paper. This shows that any NER for database and software names must be able to capture newly introduced resources to achieve high recall.

**Table 5:** Evaluation results on the development and test corpora

Development corpus	Recall (%)	Precision (%)	F-score (%)
Dictionary	49 (47)	38 (37)	43 (41)
CRF with post-processing	58 (52)	76 (67)	65 (58)
CRF without post-processing	54 (49)	78 (70)	62 (57)
Test Corpus			
Dictionary	46 (44)	46 (44)	46 (44)
CRF with post-processing	60 (54)	83 (74)	70 (63)
CRF without post-processing	53 (45)	71 (65)	62 (53)

Strict scores provided in brackets

*P* Precision, *R* Recall, *F* F-score evaluation on the development (5-cross validated) and test corpora

We note here the high variation in the different fold scores (e.g., see the results for Fold 3 in Table 6), indicate how challenging detection of resource names could be, depending on the particular document. We also note a difference between the results reported here (lenient F-score of 43–46 %) and those we obtained previously [19] on a subset of 30 documents from the development set (lenient F-score of 54 %). The drop in performance can be partially contributed to the changes to both the dataset (60 vs. 30 articles) and the underlying dictionaries (updated), as well as the change in the evaluation approach (“cross-fold” vs. evaluating the entire dataset at once; thus, a fold with an overrepresentation of false negatives cannot be balanced out by another fold with an overrepresentation of true positives (and the same for false negatives)).

**Table 6:** Dictionary matching results on the development corpus

Fold	Recall (%)	Precision (%)	F-score (%)
1	46 (43)	41 (39)	43 (41)
2	34 (31)	37 (34)	36 (32)
3	36 (34)	24 (23)	29 (27)
4	55 (53)	46 (45)	50 (49)
5	76 (75)	44 (43)	56 (55)
Min	34 (31)	24 (23)	29 (27)
Max	76 (75)	46 (45)	56 (55)
Mean	49 (47)	38 (37)	43 (41)

Note that for Fold 3, a decrease in score (of about 8 % F-score) is observed if the LINNAEUS abbreviation detected is disabled. Strict scores provided in brackets

*P* Precision, *R* Recall, *F* F-score on the development set using dictionary look-up

### Machine-learning approach

The results of the application of the CRF model are presented in Table 5. With post-processing, the average F-scores of 65–70 % for lenient and 58–63 % for strict matching present a considerable improvement over the dictionary-based approach, but still leaves the task only moderately solved. Table 7 shows the results of different folds for the development corpus. It is interesting that precision was relatively high (76–83 %), while recall was notably lower (58–60 %). These results lead us to believe that the current feature set is insufficient to capture lexical variability in sentences with biomedical resource mentions. The lenient matching scores were generally higher than the strict scores (7 % on F-score, 6 % on recall and 9 % on precision), which indicates that boundary adjustment of the recognised tokens is a challenging task, similar to other biomedical NER tasks.

**Table 7:** Machine learning results with post-processing on the development corpus

Fold	Recall (%)	Precision (%)	F-score (%)
1	51 (44)	71 (60)	59 (51)
2	44 (35)	88 (71)	59 (47)
3	51 (44)	76 (66)	61 (53)
4	65 (60)	73 (67)	69 (63)
5	80 (76)	74 (70)	77 (73)
Min	44 (35)	71 (60)	59 (47)
Max	80 (76)	88 (71)	77 (73)
Mean	58 (52)	76 (67)	65 (58)
Micro Avg	56 (50)	76 (67)	65 (57)

Strict scores provided in brackets

*P* Precision, *R* Recall, *F* F-score on the development set using machine learning with post-processing (5-cross fold)

The application of the ML-model with post-processing showed positive effects, as the results without post-processing had consistently lower recall (drop of 4–7 % for lenient and 3–9 % for strict matching). While the effect on precision was not stable, the overall F-score has still increased (3–8 % for lenient and 1–10 % for strict matching). Table 8 presents the details on the folds for the development corpus. To further evaluate the loss in recall when the post-processing step is omitted, we analysed the full list of false negative mentions to extract what percentage of these were dictionary matches, but had nevertheless been rejected by the ML approach. It turns out that this occurred in 158 (15 %) of the false negative mentions. While providing more training data could help, this issue could perhaps be also addressed by using additional features (for example, utilising some of the rules we suggest in the next section), or by combining dictionary and ML-methods. We note, however, that the direct merge of the dictionary and ML results is insufficient due to the large number of false-positives that dictionary matching introduces (see Table 9). Specifically, combining both results gives an average increase in recall of 5 % (across all folds), but a large reduction in precision, resulting in an average reduction in F-score of 15 %.

**Table 8:** Machine learning results without post-processing on the development set

Fold	Recall (%)	Precision (%)	F-score (%)
1	46 (41)	78 (69)	58 (51)
2	42 (35)	89 (75)	57 (48)
3	45 (41)	75 (70)	56 (52)
4	60 (55)	71 (66)	65 (60)
5	76 (74)	74 (72)	75 (73)
Min	42 (35)	71 (66)	56 (52)
Max	76 (74)	89 (75)	75 (73)
Mean	54 (49)	78 (70)	62 (57)
Micro Avg	52 (47)	77 (70)	62 (56)

*P* Precision, *R* Recall, *F* F-score on the development set using machine learning without post-processing (5-cross fold). Strict scores provided in brackets

**Table 9:** Combined dictionary and machine learning results on the development set

Fold	Recall (%)	Precision (%)	F-score (%)
1	56 (49)	43 (38)	49 (42)
2	50 (41)	45 (37)	48 (39)
3	57 (52)	32 (29)	41 (37)
4	68 (64)	45 (42)	54 (51)
5	87 (84)	45 (43)	59 (57)
Min	50 (41)	32 (29)	41 (37)
Max	87 (84)	45 (43)	59 (57)
Mean	64 (58)	42 (38)	50 (45)

*P* Precision, *R* Recall, *F* F-score on the development set combining the dictionary and machine learning annotations (5-cross fold). Strict scores provided in brackets

### Feature impact analysis for the ML model

We explored the impact that particular groups of features have on the recognition of biomedical resource names. During the 5-fold cross validation, each of the feature groups was removed and the CRF models were then trained and applied to the test fold enabling us to evaluate the contribution of each group. The CRF models were built without post-processing as we wanted to avoid the contributions being biased by that step (especially because it uses the dictionary predictions). The results are presented in Table 10.

**Table 10:** Feature impact analysis of the machine learning model without post-processing on the development set

Feature group	Recall (%)	Precision (%)	F-score (%)
All features	54 (49)	78 (70)	62 (57)
No lexical features	46 (43)	68 (62)	54 (50)
No syntactic features	53 (48)	77 (69)	61 (55)
No orthographic features	48 (43)	70 (62)	55 (50)
No dictionary features	49 (44)	70 (62)	57 (51)

*P* Precision, *R* Recall, *F* F-score feature contribution results comparison. Strict scores provided in brackets

Overall, the lexical features were beneficial: when this group of features was removed, there was a drop of 8 % in precision, 6 % in recall, resulting in a 7 % lower F-score. The syntactic features had only a slight impact on the performance: removing this group resulted in a 1 % drop in both precision and recall and a 2 % in F-score. The orthographic features had a similar effect as the lexical features: when these were removed, there was an 8 % loss in precision, a 6 % loss in recall, resulting in a 7 % loss in F-score. Surprisingly, removing the dictionary features did not result in a high decrease in performance (there was a drop of 8 % in precision, a 5 % drop in recall and thus a 6 % drop in F-score), suggesting that the ML-model (without the aid of a dictionary), even with the relatively limited amount of training data, managed to capture a significant number of resource mentions.

### Missed database and software mentions

We further analysed the database and software names not picked up by our ML approach for any common textual clues and patterns. Table 11 summarises different clue categories and their potential relative contribution to the overall recall. Overall, using all clues that we have recognised (see below), final recall could be as high as 94 % (Table 11), though utilising all of these pointers will likely have a detrimental effect on precision.

**Table 11:** Types of textual patterns and clues for identification of database and software names

Type	Contribution to total TPs
Machine learning matches	55.3 %
Heads and Hearst Patterns	9.8 %
Title appearances	0.5 %
References and URLs	1.8 %
Version information	0.9 %
Noun/verb associations	21.4 %
Comparisons	4.0 %
Remaining	6.3 %

Tables 12, 13, 14, 15, 16 and 17 each provide examples of the above classes

The first type of clue that seemed most discriminatory was to associate potential names with *head* terms, i.e., terms that are explicit designators of the type of resource. In the most basic case, a resource name could include a head term or be immediately followed by one (see Table 12). Key head terms included *database*, *software*, *tool*, *program*, *simulator*, *system*, *library* and *service*. Still, we note that not all potential clues are fully discriminatory. For example, we note that including *system* as a head clue might be problematic as the word can have other uses and meaning within biology (e.g., biological *systems*). Similarly, although *module* could be a useful head for identification of software names, the mention of *module(s)* in “*P and D modules”* (PMC1664705) refers to protein modules rather than programming ones. Following from this, applying standard Hearst patterns [44] could be used to extract new and unknown names from enumerations that contain some known database and software names (see Table 12). These patterns could help increase total recall by up to 10 % (Table 11).

**Table 12:** Example clues and phrases appearing with specific heads or in Hearst patterns

… the stochastic **simulator** *Dizzy* allows …
The *MethMarker* **software** was …
… **tools**: *CLUSTALW*, …, and *MUSCLE*.
… **programs** such as *Simlink*, …, and *SimPed*.

Database and software names are in italics, the associated clue is in bold

We further explored a pattern within paper titles where the papers were introducing a new resource [45]. The title would typically name the new database or software, and then follow it by a brief description (see Table 13 for examples). In the development corpus, 15 of the 60 papers (25 %) contained such a pattern that included a resource name. However, three additional papers matched the pattern, but appeared to be introducing an algorithm/method, rather than a resource. Although this would provide a limited improvement to recall on a mention level (<1 %), it could significantly aid document level recall. In addition, it provides a way to discover new tool names for inclusion in a dictionary with a high discriminatory rate.

**Table 13:** Example phrases from title appearances

*CoXpress*: differential co-expression in gene expression data
*TABASCO*: A single molecule, base-pair resolved gene expression simulator
*SimHap GUI*: An intuitive graphical user interface for genetic association analysis

Database and software names are in italics. Notice that in each case, the name is given as the initial part of the paper’s full title (preceding the colon)

Another clue is that database and software mentions are frequently followed by either a reference or a web URL (e.g., “*Galaxy* [18] and *EpiGRAPH* [19]”; PMC2784320). This was the main indicator used by OReFiL [17]. We recognise, however, that web URLs and citations are not only used for resources, and so this is far less reliable than the previous options (for example, this approach could incorrectly capture “The *learning metrics principle* [14, 15]”; PMC272927). Restricting this clue to a paper’s Methods section may reduce the potential impact on precision.

Numerous database and software mentions also contain or are accompanied by version information (see Table 14). While version numbers can be unambiguous (e.g., having ‘*v*’ or ‘*version’*), they can also be a series of numbers, which are not discriminatory enough alone (e.g., “*AMD Athlon* 1.8 GHz processor” (a CPU; PMC2242808), or “sites of *Myc* (0.22) and *NF-kappaB* (0.103)” (genes; PMC2246299)).

**Table 14:** Example versioning clues

… using *dot* **v1.10** and *Graphviz* **1.13(v16)**.
*CLUSTAL W* **version 1.83**
*Dynalign* **4.5**, and *LocARNA* **0.99**

Database and software names are in italics, the associated clue is in bold

The category with the highest potential contribution (over 21 %) includes cases where some expression (could be a noun or a verb) in the sentence (not necessarily next to the mention) gives an indication that a database or software is being referred to. Such clues can range from the more discriminatory like *website*, *screenshot* and *download*, to medium ones like *RAM*, *implement*, *simulate* and *running time*, to weak ones such as *run*, *generate*, *evaluate* and *obtain* (see Table 15 for examples). However, this type of contribution is also the one with the highest degree of variability, as many other “things” (non-database/software names) can, for example, be *run*, *implemented* or *generated*. Thus, these clues can be the most challenging to automatically and correctly associate with the actual potential resource mention. Despite some of these clues being relatively weak, we think that they have limited ambiguity at least within the field of bioinformatics, even if this is not true in a different field. To roughly estimate the effect on precision that inclusion of these clues may have, we compared the number of sentences in the development corpus with a specific clue from this category to the number of sentences with both the clue and a database or software name within the corpus. For example, 76 % of sentences which matched the word *website* also contained a resource mention, while only 50 % of sentences that matched *RAM* contained a mention of database or software. However, despite our assumption that “*to run*” (in any verb form) was a (relatively) good indicator, it actually appears to have low correlation with resource names, as only 11 % of sentences which matched the regular expression “ran|run(ning|s)?” also contained a resource mention (however, 8 % of sentences which contained a resource mention also matched that regular expression). Nevertheless, there could still be merit in these clues if used in combination with each other rather than alone.

**Table 15:** Example expressions that functionally indicate database and software mentions

… the *SimHap GUI* **installation**.
… **implemented** within *PedPhase* …
*MethMarker* therefore **provides** …
A typical **screenshot** of *MethMarker* …
*Cofolga2* has six free **parameters** …
*MethMarker’s* **user interface** reflects …
*MethMarker* can directly **import** …
*xPedPhase* thus needs **cubic time** …

Database and software names are in italics, the associated clue is in bold

A number of clues can be inferred from sentences that make some comparison between two or more database and software names (see Table 16). Many of these examples can be considered as extended Hearst patterns (e.g., “like *tool1*, *tool2* is …”) but we have analysed them separately for a couple of reasons. In particular, there are an unusually high number of terms contained within this class in the development corpus (although, a third of the examples within this class all come from a single paper). Following on from this, in most of the cases within this class, neither resource being compared in each case was present in our dictionary. Thus, even if the comparison pattern has been implemented, the method would need at least to know about some of the tools to infer others. As such, although we envisage potential in addressing this type of database and software mention, we cannot extrapolate how much use it could have due to the biased sample.

**Table 16:** Examples of comparisons between database and software names

… the numbers of breakpoint sites by *xPedPhase* were **equal to** the numbers of breakpoints by *i Linker*…
*xPedPhase* **did better than** *i Linker*…
*Cofogla2* with this cutoff PSVM gives a better false positive rate **compared to** *RNAz*…
*Foldalign* was much **slower than** *Cofolga2* except for…
**Like** *Moleculizer*, *Tabasco* dynamically generates…

Database and software names are in italics, the associated clue is in bold

Finally, there are a series of mentions (around 6 %) without any clear textual clue, or with particularly ambiguous ones (see Table 17 for examples). Some potential clues such as *analyse*, *contains*, *column*, *step* and *matrix* seem too generic within the bioinformatics field to be useful. For example, the number of sentences within our corpus that contained both the regular expression “analyse(d|s)?|analysis” and a mention of a database or piece of software was only about 21 %, whereas it was even lower for the regular expressions “step(s|ped)?” (14 %) and “contain(ed|s)?” (13 %).

**Table 17:** Example phrases with no clear or discriminative clues

Additionally, *i Linker* has an error correction step that detects unlikely crossover events.
In addition, *Tabasco* should be a good base to further study interactions on DNA…
*PSPE* is not only able to use one of many common models of nucleotide substitution…
The results show that *LibSELDI* tends to have a considerable advantage in the low FDR region…
The structure of *Tabasco* confers at least four advantages.

Database and software names are in italics

### False positive filtering

Some typical false positive mistakes returned by the CRF models include mentions of programming languages and their libraries (e.g., *Python*, *BioPython*), algorithms/methods (e.g., *Euclidean* – a distance measure, *BLOSUM* – a similarity scoring matrix), file formats (e.g., *FASTA*), companies and organisations (e.g., *EBI* – the *European Bioinformatics Institute*). While we have explicitly excluded these types from the current task, they can still be useful indicators of bioinformatics practice. Another large class of errors, like with the dictionary approach alone, is with matches of *GO* sub-string within database identifiers (e.g., *GO:0007089*). Finally, ambiguous acronyms are typically returned as errors, but could be checked by searching for a definition within the document.

We note that there is not always a clear distinction between database and software names, methods, approaches, algorithms, programming languages, database records/identifiers, and file formats. We have decided to focus on “executables” and datasets as our ultimate aim is to help reconstruct the bioinformatics workflow that has been used within a given paper, so that we can support experiment replication and reproduction. The problem occurs because authors often introduce a novel algorithm and associated implementation (e.g., as a service or a stand-alone application), but frequently refer to their contribution only as an algorithm (or method), rather than software (or vice-versa). As such, although they are talking about their algorithm throughout the paper, it could be argued that they are referring to their software implementation, especially when talking about benchmark improvements in results. The fuzzy boundary between these definitions is a challenge for any focused automated system to overcome. Still, this distinction may not be relevant for some applications.

## Conclusions

In this paper we presented an exploration of variability and ambiguity of database and software mentions in the bioinformatics and computational biology literature. Our results suggest that database and software NER is a non-trivial task that requires more than just a dictionary matching approach, even when using comprehensive resource inventories. Due to bioinformatics’ focus on resource creation, a dictionary would never be sufficiently comprehensive, making resource recognition potentially as hard as gene recognition (in contrast to species recognition, which is a relatively stable domain). Example names such as *Network* and *analysis* provide sources of ambiguity, whereas acronyms and verbalised references to software such as *BLASTed* provide issues of variability that need to be overcome.

The results of our ML-model show that dictionary-based predictions can be significantly improved. While ML achieved a major increase in precision, boosting recall proved to be challenging, indicating that additional attributes need to be included for accurate biomedical resource recognition.

Our analyses also provided a series of clues that could be picked up by text-mining techniques. As many of these clues are ambiguous on their own, an approach would be to combine various evidence (e.g., using voting and threshold) in order to capture database and software names more accurately (see, for example, [8]). Further work could combine these rules with the machine learning system to further increase the overall system accuracy, perhaps helping to recover some of the lost recall.

### Availability of supporting data

The datasets supporting the results of this article are available at: http://sourceforge.net/projects/bionerds/.

## Abbreviations

IAA: Inter-annotator agreement
BLAST: Basic local alignment search tool
CRF: Conditional random fields
GO: Gene ontology
ML: Machine learning
NER: Named entity recognition
PDF: Portable document format
PMC: PubMed central
POS: Part-of-speech
SCOP: Structural classification of proteins
